# Ion Permeation Mechanism in Epithelial Calcium Channel TRVP6

**DOI:** 10.1038/s41598-018-23972-5

**Published:** 2018-04-09

**Authors:** Serzhan Sakipov, Alexander I. Sobolevsky, Maria G. Kurnikova

**Affiliations:** 1 Chemistry Department, Carnegie Mellon University, 4400 Fifth Ave., Pittsburgh, PA 15213 USA; 2 Department of Biochemistry and Molecular Biophysics, Columbia University, 650 West 168th St., New York, NY 10032 USA

## Abstract

Calcium is the most abundant metal in the human body that plays vital roles as a cellular electrolyte as well as the smallest and most frequently used signaling molecule. Calcium uptake in epithelial tissues is mediated by tetrameric calcium-selective transient receptor potential (TRP) channels TRPV6 that are implicated in a variety of human diseases, including numerous forms of cancer. We used TRPV6 crystal structures as templates for molecular dynamics simulations to identify ion binding sites and to study the permeation mechanism of calcium and other ions through TRPV6 channels. We found that at low Ca^2+^ concentrations, a single calcium ion binds at the selectivity filter narrow constriction formed by aspartates D541 and allows Na^+^ permeation. In the presence of ions, no water binds to or crosses the pore constriction. At high Ca^2+^ concentrations, calcium permeates the pore according to the knock-off mechanism that includes formation of a short-lived transition state with three calcium ions bound near D541. For Ba^2+^, the transition state lives longer and the knock-off permeation occurs slower. Gd^3+^ binds at D541 tightly, blocks the channel and prevents Na^+^ from permeating the pore. Our results provide structural foundations for understanding permeation and block in tetrameric calcium-selective ion channels.

## Introduction

Ion channels in biological membranes conduct ions with speeds approaching diffusion limit (often faster than 10^6^ ions per second)^1^. Ion channels are narrow water-filled pores formed by the membrane inserted proteins connecting two sides of the lipid bilayer. An amazing speed of the ion transport often co-exists with high pore selectivity for one ion type over the others. Better known examples of such protein pores include tetrameric K^+^ channels that can conduct potassium more than 10^4^ times better than sodium, and Na^+^ channels^2,3^. The structural organization of the K^+^ channel pore was uncovered using crystallography^4,5^, while detailed understanding of permeation mechanism was greatly facilitated by numerous molecular dynamics (MD) and quantum mechanics (QM) studies^2,6–18^. Selectivity of specialized sodium channels to sodium was explored using the bacterial Na^+^ channel Na_V_Ab as a template^2,19–24^. Recent improved understanding of the mechanisms of monovalent ion permeation has been progressing in parallel with rapid advances in computational structural biology. Improved computational efficiency as well as advancement in models of molecular interaction (the so called force-fields) now allow for realistic modeling of ion channel proteins and their interaction with a variety of ions^25–29^ including divalent ions. In some cases, advanced theoretical approaches of polarizable force-fields^30^ and Quantum Mechanical methods^31^ are now applicable to protein systems. In this study we employ these modern techniques to study interaction of ions with a calcium selective channel.

Unlike monovalent ion-selective channels, calcium-selective ion channels are studied less well, despite unique and ubiquitous role of Ca^2+^ as a signaling molecule contributing to numerous physiological processes and diseases^32–34^. The lack of high resolution structural information on architecture of Ca^2+^-selective channels is the main culprit to prevent such studies. Only in 2012, the first structure of a calcium-selective ion channel, a hexameric calcium release-activated current (CRAC) channel Orai has been solved^35^. However, structural information about calcium permeation in tetrameric calcium-selective ion channels, a large and diverse family of physiologically and medicinally important proteins^36^, has been missing until recently. Cryo-EM reconstructions of Ca_V_1.1 were the first resolved structures in this class^37,38^. However, due to low resolution and inability of cryo-EM to reveal identity of ions in the pore, these structures provided little insight into calcium permeation mechanism. An artificial protein Ca_V_Ab, a bacterial Na^+^ channel genetically engineered to become a calcium-selective channel, was crystallized and provided the first glimpse into calcium selectivity of tetrameric ion channels^39^. Due to the lack of high resolution structural information on Ca^2+^ selective proteins, computational modeling and analysis of divalent ion permeation consequently remains in the realm of mostly simplified model systems^31,40–47^.

We have recently solved the first crystal structures of a eukaryotic tetrameric calcium-selective ion channel TRPV6^36^ that plays a vital role in calcium homeostasis as a Ca^2+^ uptake channel in epithelial tissues and is implicated in a variety of human diseases, including cancers^48–56^. These structures resolve the ion channel selectivity filter in the presence of several ions, including Ca^2+^, Ba^2+^ and Gd^3+^ and represent the first naturally occurring molecular template of a calcium specific tetrameric channel that can now be analyzed to develop the mechanism of calcium permeation and understand calcium selectivity in Ca^2+^ channels. The present study develops the mechanism of calcium permeation in TRPV6 using MD and quantum mechanics modeling. It also presents an analysis of Ba^2+^ permeation and Gd^3+^ block of the channel. Our model is the first one in which permeation of a divalent ion through the selectivity filter is clearly demonstrated in a fully resolved structure of a Ca^2+^-selective protein. A recently reported simulations study of the TRPV1 channel, a protein of the same family as TRPV6, did not resolve Ca^2+^ permeation mechanism^45,46^.

**Figure 1:**
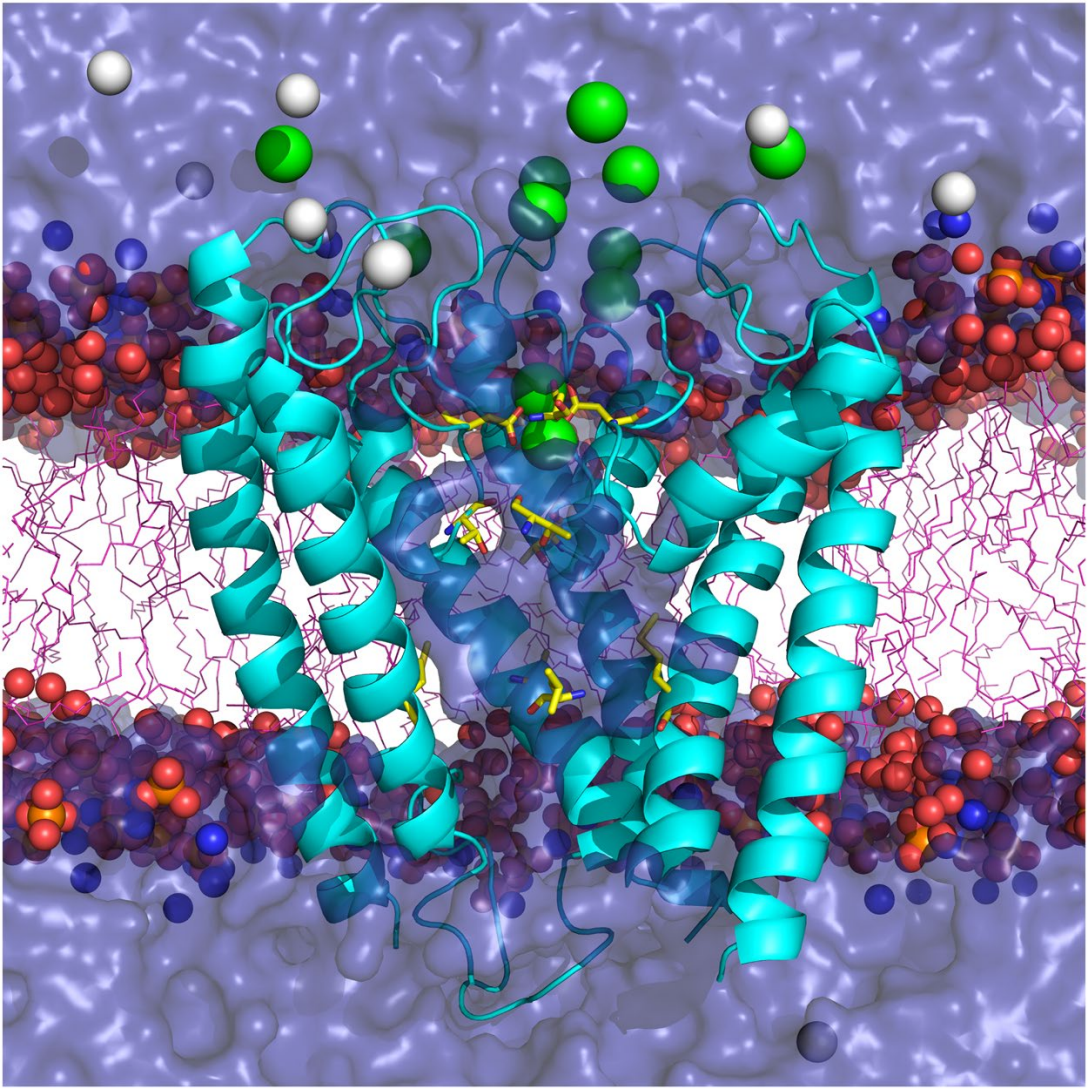
TRPV6 simulated system. Truncated TRPV6 (see Methods) is shown in lipid bilayer and water in the presence of Na^+^ and Ca^2+^ ions. For all simulations, composition of protein/lipid/water remained the same, while composition of ions varied (see text). The model protein consisted of the pore domain (S5, P-loop, and S6) and a truncated TRP helix (residues K483 to Q595, not shown). The protein (cyan) is shown in cartoon representation. Pore lining residues D541, T538, and M569 are shown as yellow sticks. The POPC lipid head-groups are shown as colored spheres: oxygen is red, phosphorus is orange, and nitrogen is blue. The hydrocarbon lipid tails (pink) are shown in stick representation. Ca^2+^ and Na^+^ ions are shown as green and white spheres, respectively, and water is illustrated by a semi-transparent continuum (blue).

To uncover the molecular mechanism of calcium permeation, we constructed a system of the TRPV6 channel embedded in lipid bilayer and water (Fig. 1), which was stable in our MD simulations. We observed ion behavior in the channel at equilibrium and in conditions conducive to ion permeation through the selectivity filter. We have further confirmed predictions deduced from MD simulations using advanced quantum chemical approach. Consistent with previous physiological studies^43^, we found that Na^+^, Ba^2+^, and Ca^2+^ ions permeate through the channel, while Gd^3+^ does not. We also confirmed that interactions of metal ions with aspartates D541 play a key role in Ca^2+^ selectivity. Our major finding is the direct demonstration of the knock-off mechanism of Ca^2+^ permeation previously proposed by Saotome *et al*.^36^. Our findings set firm ground to describe principles of calcium selectivity in tetrameric ion channels and create foundations for future modeling studies.

## Results

### Equilibrium Simulations

Equilibrium MD simulations of the TRPV6 channel were performed in conditions closely related to the physiological, i.e. at room temperature and in presence of the lipid membrane, water and ions. Figure 1 shows an overview of a typical simulated system (see Methods for the detailed description of the simulated systems and protocols). Equilibrium MD trajectories of TRPV6 in its Apo form and in presence of ions Ca^2+^, Ba^2+^ or Gd^3+^ (named CA, C1, B1 and G1 respectively; see Methods, and Table ST1) were initiated with the protein and ion coordinates determined by the corresponding crystal structures (PDB IDs: 5IWK, 5IWP, 5IWR and 5IWT). Since in all these structures the ion channel is closed, permeation of ions was studied at the selectivity filter and not expected to occur through the S6 helices bundle crossing gate at M577.

**Figure 2:**
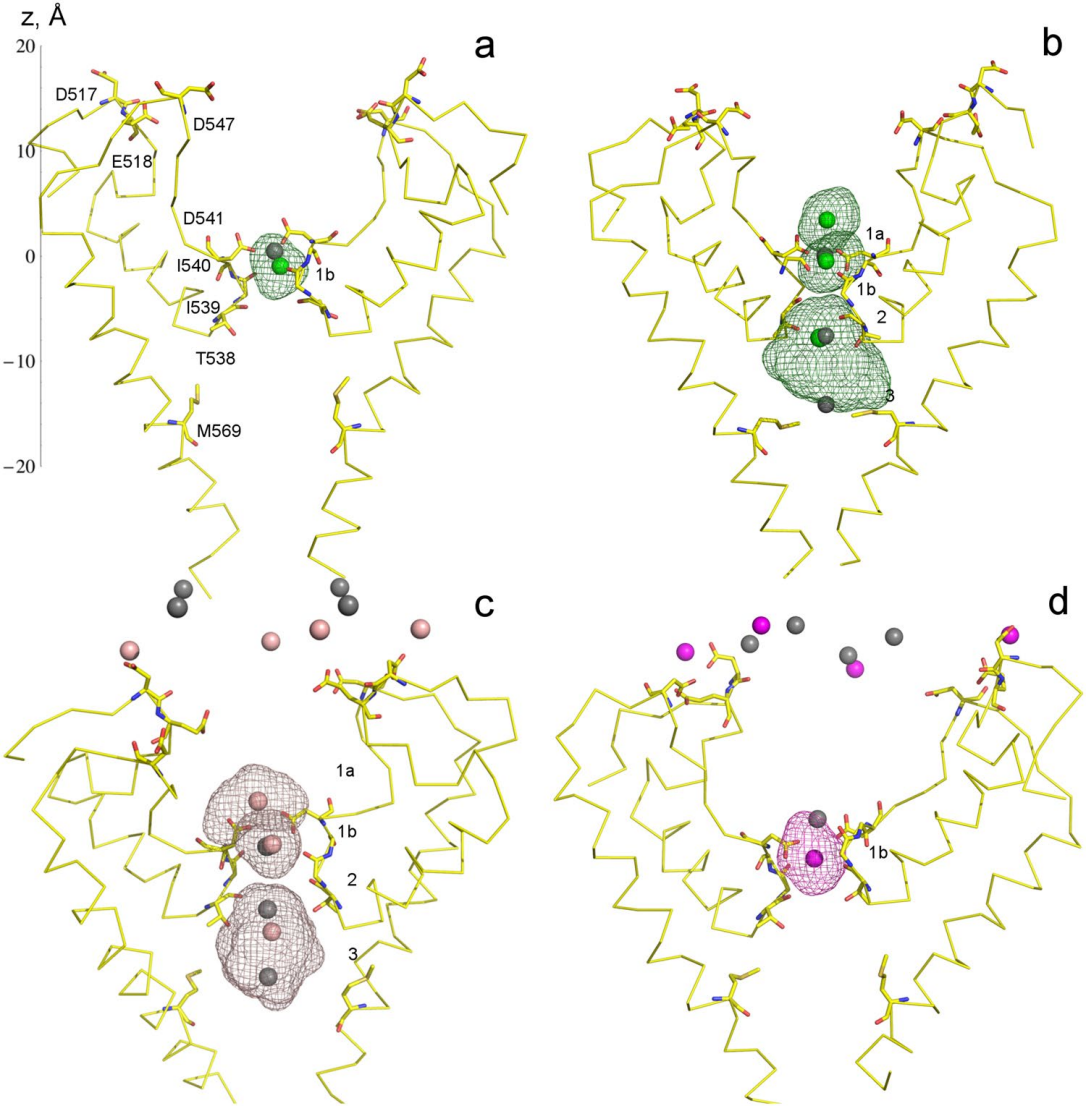
Equilibrium ion positions in TRPV6 channel. Colored spheres show MD-predicted positions of Ca^2+^ (green), Ba^2+^ (peach), and Gd^3+^ (magenta), while the crystal structure-predicted positions are shown as grey spheres. Each simulation was initiated with multivalent ions positioned as in the corresponding crystal structures, and the monovalent ions (not shown) were placed randomly to satisfy electrostatic neutrality of the simulated system. The protein is shown in yellow, oxygen atoms are red and nitrogen atoms are blue. Water and lipid molecules are not shown. The volumes indicated by mesh surfaces represent spaces occupied by ions during the simulations. (**a**) A single bound Ca^2+^ ion in CA simulation. The z = 0 position along the z-axis corresponds to Site 1 coordinate in the crystal structure. **(b)** Ca^2+^ ion positions in C1 simulation. **(c)** Ba^2+^ ion positions in B1 simulation. **(d)** Gd^3+^ ion positions in G1 simulation.

In our simulations, Ca^2+^, Ba^2+^, and Gd^3+^ ions occupied similar but not identical pore positions compared to the crystal structures and designated as Sites 1–3 (Fig. 2). We retain this crystal structure nomenclature of ion binding sites and, when necessary, amend it with newly identified positions (see below). The single Ca^2+^ ion bound to the Apo state crystal structure (PDB 5IWK) remained close to its original Site 1 position in the CA simulation (Fig. 2a). The T538-bound Ca^2+^ ion located at Site 2 in the crystal structure obtained at high calcium concentration (PDB 5IWP) shifted towards Site 1 associated with D541 in the corresponding simulation C1 (Fig. 2b). Therefore, crystallographically identified Site 1 is occupied by two Ca^2+^ ions at positions designated as Sites 1a and 1b. The third Ca^2+^ ion in the pore remained at the lower binding Site 2^36^ but was fairly mobile during the simulation, sampling positions that also include the Site 3 (see the wireframe representation of the trajectory in Fig. 2b).

In order to further verify the MD-predicted Ca^2+^ Sites 1a and 1b, we performed high level *ab initio* quantum mechanical (QM) energy calculations (see Methods). We compared potential energy of the crystal-like ion configurations and equilibrium configurations observed in MD simulations (Fig. S1). These calculations included 620 structures extracted from the C1 trajectory, each containing two Ca^2+^ ions at Site 1 with their first coordination shell ligands (D541 and nearest waters), and partial charges of the outer environment as described in Methods and shown in Fig. S1. The average ab initio energy of the ion configurations produced in MD simulations was 39.5 kcal/mol lower than the average energy of the crystal-like configurations. While the absolute potential energy of a cluster in ab initio calculations cannot be compared directly with any of the experimentally measured energies for such system, the high absolute energy difference of ab initio computed cluster energies is indicative of the preference for such clusters in the full system. This result strongly supports feasibility of the MD predicted ion binding site configurations in the TRPV6 pore.

Stable positions of the Ba^2+^ ions during the equilibrium B1 trajectory (PDB 5IWR) are shown in Fig. 2c. Equilibrium positions of Ba^2+^ ions in the pore are similar to the positions of Ca^2+^ ions in the trajectory C1 (Fig. 2b). Ba^2+^ ions located at the recruitment sites formed by D517, E518 and D547 in the ion channel extracellular vestibule^36^ remained at the corresponding positions throughout the simulation. The trajectory G1 was initiated with Gd^3+^ ions placed at positions identified by the crystal structure obtained at high gadolinium concentration (PDB ID: 5IWT). In this simulation, the Gd^3+^ ion originally positioned at Site 1 shifted towards the interior of the selectivity filter to occupy Site 1b (Fig. 2d). This shift resulted in a stable coordination of the corresponding Gd^3+^ ion by one of the D541 residues and the backbone carbonyl oxygens of residues I540 and I539 in the selectivity filter.

**Figure 3:**
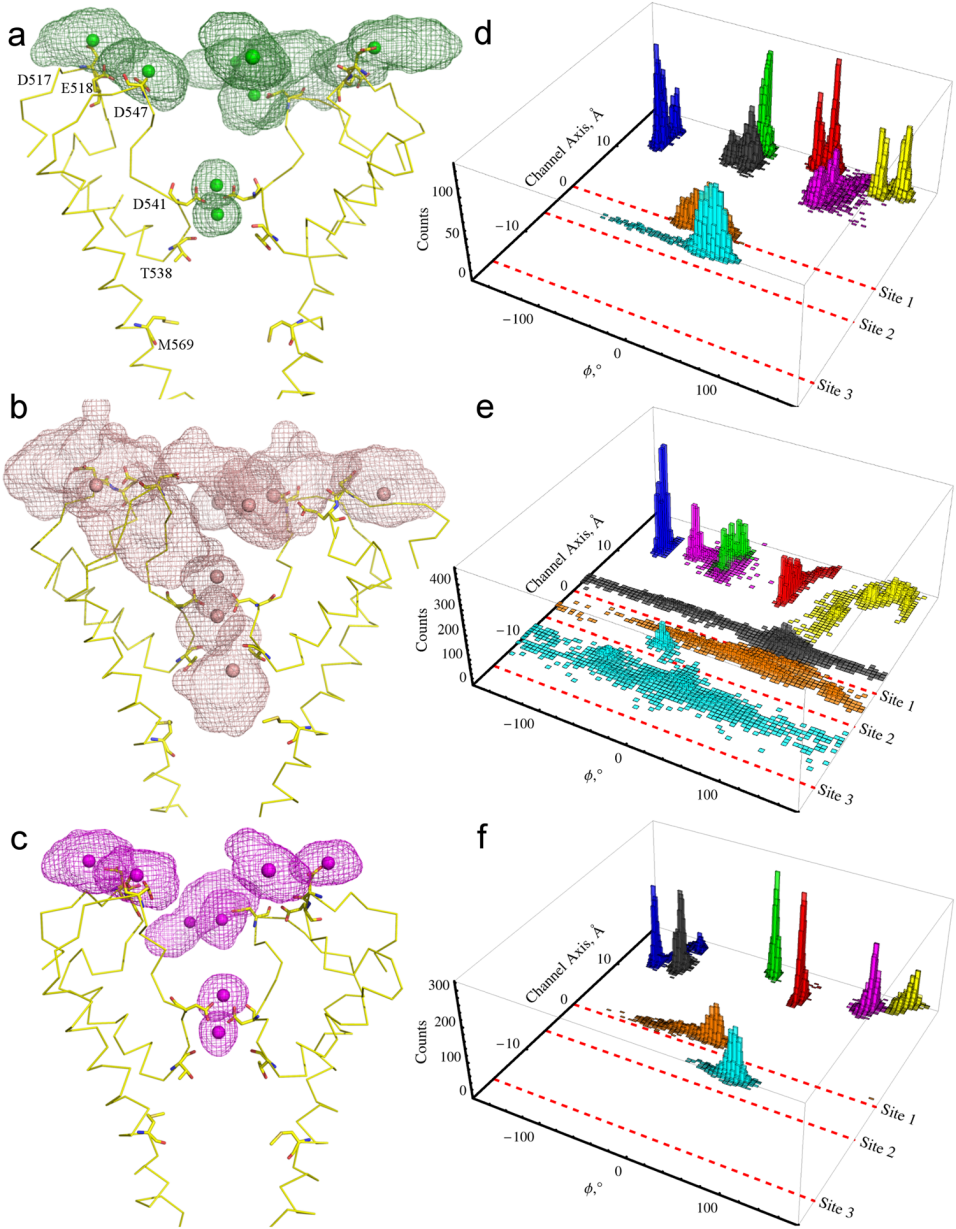
Preferred positions of multivalent ions in long equilibrium simulations. (**a**–**c**) Space occupied by Ca^2+^ during C5 (**a**), Ba^2+^ during B2 (**b**) and Gd^3+^ during G2 (**c**) 110 ns-long trajectories are shown as green, peach, and magenta meshes, respectively. **(e–f)** Ion distributions in the C5, B2 and G2 simulations respectively in 2D coordinates: the distance along z-axis illustrated in Fig. 2a and the polar angle in a plane perpendicular to the pore axis at z = 0. Red, green, blue, and yellow histograms represent individual ions positioned at the recruitment sites. Black and magenta histograms represent two ions located elsewhere in the channel vestibule. Orange and cyan histograms represent two ions located at the pore sites. Red dashed lines indicate ion binding sites predicted by crystal structures at D541 (Site 1), T538 (Site 2), and M569 (Site 3).

The relative mobility of Ca^2+^, Ba^2+^, and Gd^3+^ ions observed in the long trajectories C5, B2 and G2 that have similar composition of multivalent ions is shown in Fig. 3. All ions located at the recruitment sites are more mobile than the ions bound to the D541 site. Ca^2+^ and Gd^3+^ ions initially bound to the D541 Sites 1a and 1b remained in their positions. In contrast, Ba^2+^ ion initially bound to Site 1b permeated through the pore into the central cavity, to Sites 2 and 3, after another ion, from the recruitment site, left its initial position and approached the D541 Site 1a. Ca^2+^ and Gd^3+^ ions located in the channel vestibule (black and magenta bars in Fig. 3d–f) diffused towards the recruitment sites and remained relatively stable. Judging by the sharpness and height of the histogram peaks, Gd^3+^ appears to be relatively more stable than Ca^2+^ in the recruitment sites. The yellow peak representing one of Ba^2+^ ions at the recruitment sites has spread out towards the Site 1a.

### Non-equilibrium Simulations

To understand the mechanism of selective ion permeation in the TRPV6 channel, we designed multiple non-equilibrium MD simulations that began with no resident ion in the central cavity of the channel (as well as no ions on the extracellular side of the membrane). The ion concentration gradient created between the channel vestibule and the central cavity thus served as a driving force for the ions to permeate through the selectivity filter into the water filled central cavity of the channel. The simulations presented in the following sections were stopped when a permeation event occurred, or, when a permeation event did not occur in 30 ns (chosen to be sufficiently longer than the permeation event itself, see below).

#### Na^+^ and Ca^2+^

**Figure 4:**
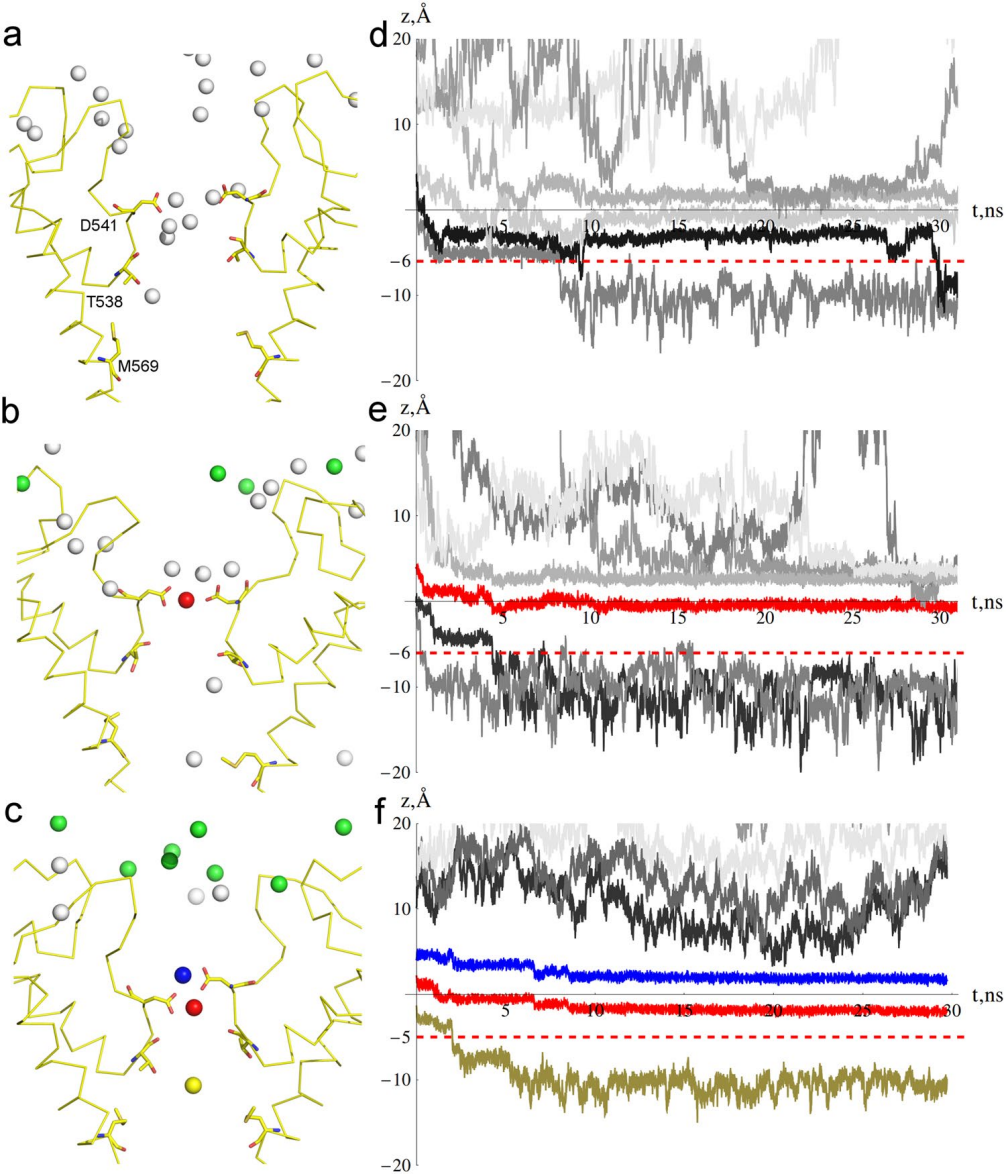
Ca^2+^-dependent Na^+^ permeation through TRPV6 channel. Equilibrium positions of Na^+^ (white spheres) and Ca^2+^ (green, blue, red or mustard spheres) ions (**a**–**c**) and the corresponding ion trajectories along the channel axis (**d**–**f**). (**a**,**d**) C2 simulation of Na^+^ permeation in the absence of Ca^2+^, (**b**,**e**) C3 simulation of Na^+^ permeation at low Ca^2+^ concentration, and (**c**,**f**) C6 simulation with high Ca^2+^ concentration that features a Ca^2+^ permeation event but no Na^+^ permeation. The trajectories of individual Na^+^ ions are shown in various shades of grey, while Ca^2+^ trajectories are in the same colors as ions in (a–c). The horizontal red dashed lines (d–f) indicate the z-axis level below which an ion has successfully permeated the selectivity filter. The residues labeled in (a) are also shown in b) and c) and in the following figures.

It is known that at low Ca^2+^ concentrations, the inward current through the TRPV6 channel is mainly carried by Na^+^ ions. An increase in Ca^2+^ concentration leads to an increase in Ca^2+^ current and a reduction of Na^+^ current^57–60^. At high Ca^2+^ concentrations, Ca^2+^ current prevails, making the channel highly selective to calcium. To study the mechanism of Ca^2+^ permeation and the interplay between Ca^2+^ and Na^+^ permeation, we performed non-equilibrium MD simulations with varying number of Ca^2+^ and Na^+^ ions at around D541 and in the extracellular vestibule, including recruitment sites (trajectories C2-C7 in Table ST1). Stable positions of Na^+^ ions during the C2 simulation that did not contain any Ca^2+^ ions is illustrated in Fig. 4a. Inside the pore, Na^+^ ions coordinate D541 residues as well as backbone oxygens of T538, I529, and I540. The individual trajectories of Na^+^ ions (Fig. 4d) demonstrate that sodium freely permeates the TRPV6 selectivity filter (see illustration of the permeation process in Suppl. Movie 1). In simulations C3-C7, four Ca^2+^ ions were placed at the ion recruitment sites^36^, while the number of Ca^2+^ ions elsewhere in the extracellular vestibule and at Site 1 varied. In C3 trajectory, one Ca^2+^ was placed at Site 1 (See Figs 4b and S2a for initial and equilibrium configurations of ions in the C3 simulation). At equilibrium, two of four aspartates D541 coordinated Ca^2+^, while the other two coordinated Na^+^ ions. Sodium ions but not Ca^2+^ were able to permeate through the selectivity filter (Fig. 4b,e). In C4 simulation, two additional Ca^2+^ ions replaced Na^+^ ions at Site 1 (Fig. S2b), resulting in rapid Ca^2+^ permeation towards Sites 2 and 3 (Fig. S2b). In C5 simulation, two Ca^2+^ ions were placed at Sites 1a and 1b and two more Ca^2+^ ions in the extracellular vestibule (Fig. S2c shows the initial and equilibrium configurations of ions in C5). Two Ca^2+^ ions at D541 remained stable during the 160 ns-long C5 simulation and no Ca^2+^ or Na^+^ permeation occurred. During the equilibrium phase of C5, calcium ion at Site 1a coordinated five D541 O_δ_ atoms (carboxylate groups of the aspartate residues that form the channel constriction) and two water molecules from the channel vestibule. The Site 1b Ca^2+^ ion coordinated two O_δ_ of D541 and four water molecules from the selectivity filter (Fig. S2c).

**Figure 5:**
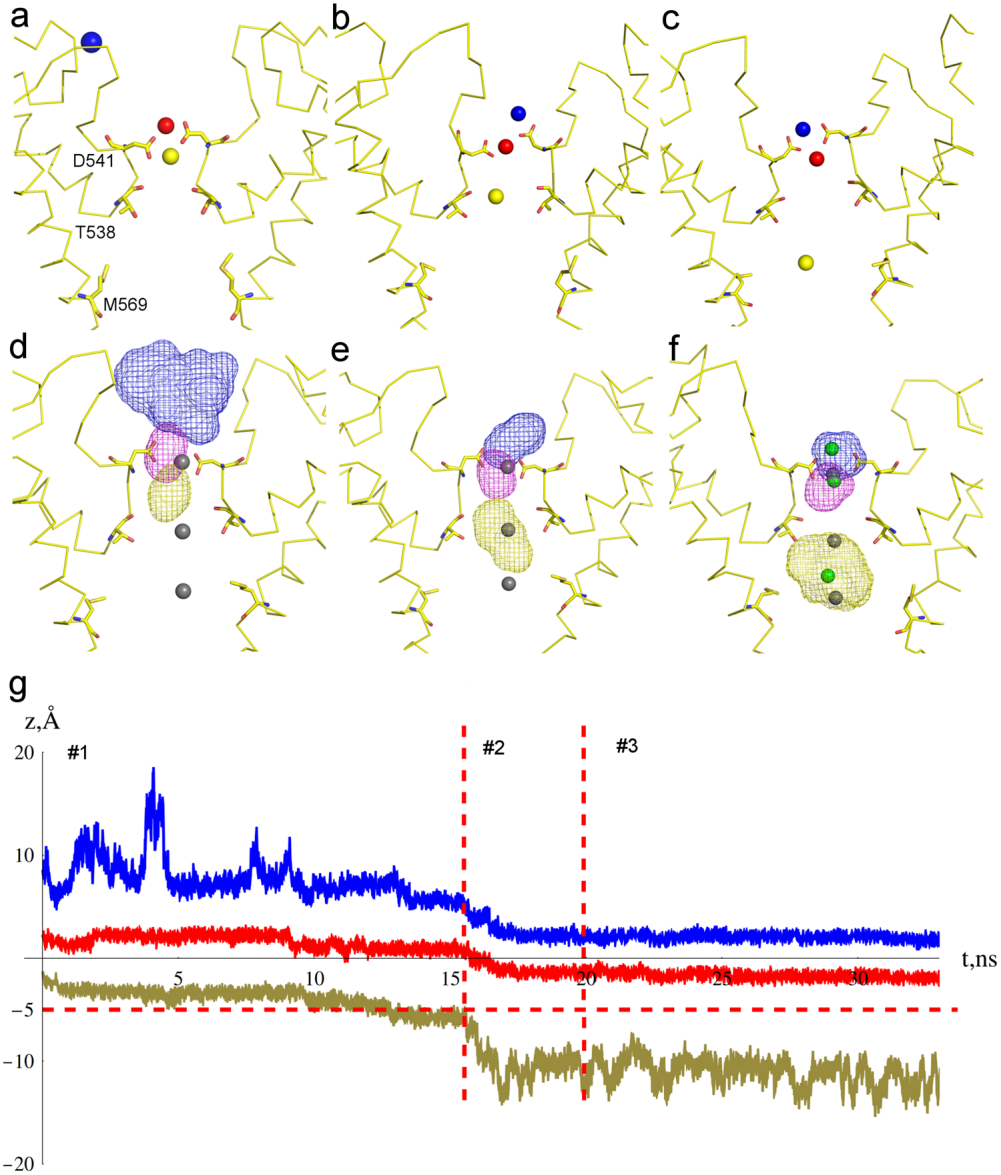
Knock–off mechanism of Ca^2+^ permeation. (**a**–**f**) Sequential representative positions of Ca^2+^ ions in the C7.1 simulation in (**a**,**d**) initial configuration, (**b,e**) transition state, and (**c**,**f**) final configuration. Blue, magenta and mustard spheres (**a**–**c**) represent incoming, intermediate and leaving Ca^2+^ ions, respectively. Similarly colored mesh surfaces (**d**–**f**) represent spaces occupied by the corresponding ions throughout the simulation. Residues D541, T538, and M569 are shown in stick representation. (**g**) Ca^2+^ ion trajectories along z-axis during C7.1 simulation. The vertical red dashed lines indicate states #1–3 illustrated in (**a**,**d**), (**b,e**), and (**c**,**f**) respectively. The horizontal red dashed line indicates the level of ion crossing into the channel.

**Figure 6:**
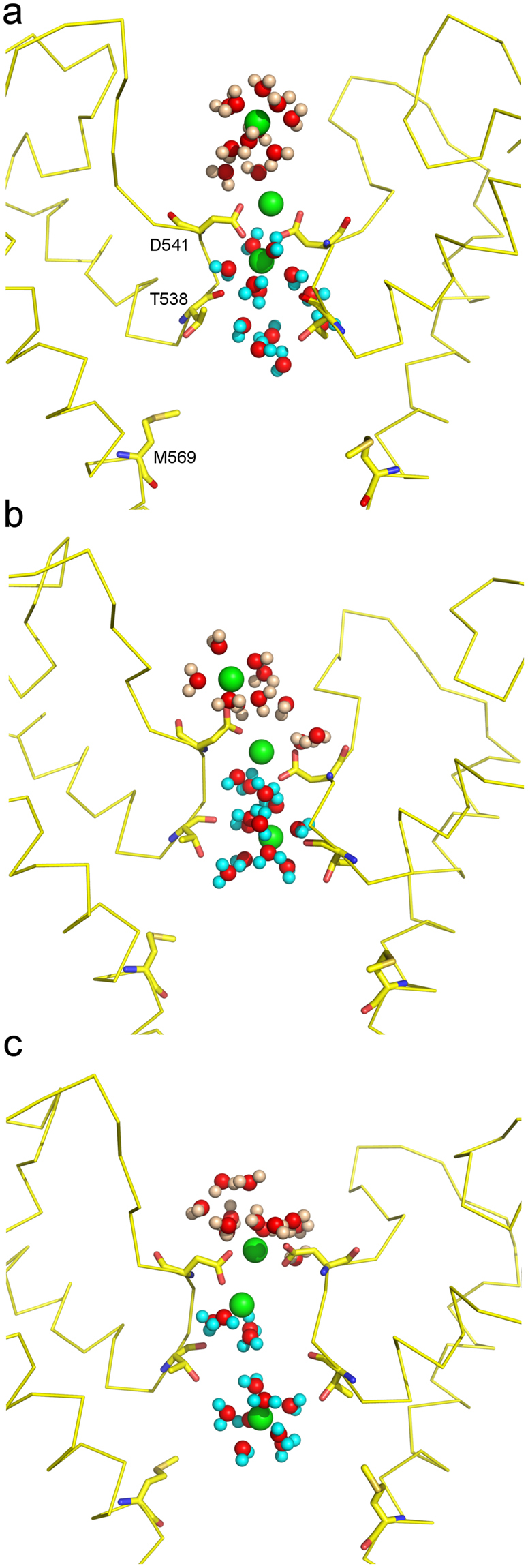
Water coordination of Ca^2+^ ions during permeation. Shown are Ca^2+^ ions and water molecules during C7.1 simulation in the (**a**) initial, (**b**) intermediate, and (**c**) final configurations illustrated in Fig. 5. Water molecules initially located externally to the D541 are shown with the pink colored hydrogens. Water molecules initially located internally to D541 are shown with the cyan colored hydrogens. Oxygen atoms are shown in red. Note, water does not permeate the selectivity filter.

Three trajectories (C6, C7.1, and C7.2) started with the total of eleven Ca^2+^ ions in the extracellular vestibule and at D541. The Ca^2+^ ions concentration gradient across the selectivity filter corresponds to a driving potential of ca. *V* = −*20* *mV*. When three Ca^2+^ ions were placed in close vicinity to Sites 1a and 1b (simulation C6) rapid permeation of the Ca^2+^ into the central cavity occurred (Fig. 4c,f). In the beginning of C7 simulations, only two Ca^2+^ ions were placed at Sites 1a and 1b and the closest calcium ion in the extracellular vestibule was placed approximately 7Å away from Site 1a. In C7.1 simulation, this closest Ca^2+^ ion approached Site 1a and the permeation event occurred via a clear knock-off mechanism with a short-living transition state (Fig. 5 and Suppl. Movie 2). No Ca^2+^ permeation occurred during C7.2 simulation. During permeation, the incoming ion, initially fully coordinated by water of the extracellular vestibule, gradually exchanged these water molecules to carboxylic oxygens of D541 (Fig. 6). Calcium ion initially bound to Site 1a remained coordinated by aspartates D541 but shifted towards Site 1b, exchanged water molecules from the vestibule to D541 O_δ_ atoms, and finally lost one D541 O_δ_ atom in exchange to water molecules of the selectivity filter. In these conditions, two Ca^2+^ ions are always present at Site 1, with no water molecules present between them and no water molecules crossing from the extracellular vestibule to the selectivity filter (Fig. 6). Similar behavior was observed for Na^+^ ions. From Site 1b calcium moves towards Sites 2 and 3 by exchanging the ligands in the first coordination shell to water molecules of the selectivity filter and central cavity.

#### Ba^2+^

In order to study Ba^2+^ permeation, we performed simulation B2 with 8 Ba^2+^ ions: four at the recruitment sites, two elsewhere in the vestibule, and two at Sites 1a and 1b. In the initial stable configuration of this simulation (Fig. S3a), Ba^2+^ at Site 1a was coordinated by two water molecules from the vestibule and six D541 O_δ_ atoms, while Ba^2+^ at Site 1b was coordinated by six water molecules from the selectivity filter, one O_δ_ of D541 and one backbone oxygen of T538. During 143-ns simulation, one permeation event occurred after a long-lived transition state with three Ba^2+^ ions at Sites 1a and 1b (Fig. S3b): one coordinating six water molecules from the vestibule and two O_δ_ atoms of D541, another coordinating three water molecules and five D541 O_δ_ atoms, and the third ion coordinating one O_δ_ of D541, one backbone oxygen of T538 and six water molecules from the selectivity filter. Figure S3d shows trajectories of ions in the pore during permeation. Notably, after the third Ba^2+^ ion approached Site 1a (blue line reached ~15-ns time mark in Fig. S3d), all three Ba^2+^ ions remained at the same positions around D541 for almost 30 ns (an apparent transition state configuration). Then, the lower ion (yellow line in Fig. S3d) lost its coordination with D541 at Site 1b and permeated through the selectivity filter (Fig. S3c).

#### Gd^3+^

**Figure 7:**
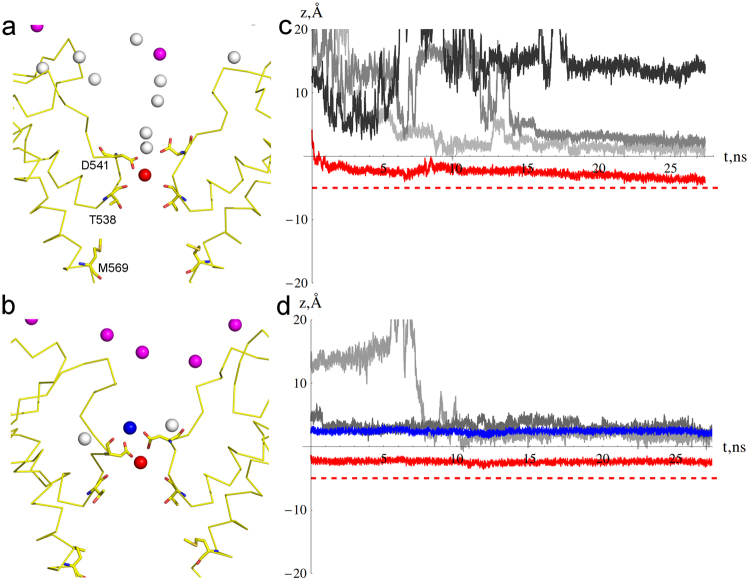
Gd^3+^ block of TRPV6 channel Equilibrium positions of Gd^3+^ (pink, red, or blue spheres) and Na^+^ (white spheres) ions (**a**,**b**) and the G1 and G2 trajectories along the channel axis (**c**,**d**) in (**a**,**c**) G1 simulation at low Gd^3+^ concentration with a single Gd^3+^ ion (red) and (**b**,**d**) G3 simulation at a higher Gd^3+^ concentration with two Gd^3+^ ions (blue and red) around D541. The trajectories (**c**,**d**) of individual Na^+^ ions are shown in various shades of grey, while Gd^3+^ trajectories are in the same colors as ions in (**a**,**b**). The horizontal red dashed lines indicates the level which would be crossed if ion permeates the channel.

To study behavior of Gd^3+^ ions in the TRPV6 pore, we run simulations G1-G4 with increasing number of Gd^3+^ ions (Table ST1). The simulation length varied between 30 ns and 160 ns. Gd^3+^ ions placed at the recruitment sites remained bound to the extracellular vestibule residues D517 and D547 (see Fig. 3f). No ion permeation was observed during simulations G1-G4. In G1, where we had the smallest number of Gd^3+^ ions, we observed a stable ion configuration with one Gd^3+^ ion at Site 1 coordinating three D541 O_δ_ atoms and six water molecules of the selectivity filter and Na^+^ ions coordinating the rest of the D541 O_δ_ atoms (Figs 7a and S4a). The 160 ns-long G2 simulation at higher Gd^3+^ concentration resulted in a stable configuration (Fig. 7b) with two Gd^3+^ ions in the selectivity filter: one ion coordinating three D541 O_δ_ atoms and six water molecules of the selectivity filter, and another one coordinating four D541 O_δ_ atoms and four water molecules from the vestibule. G3 simulation with the same total number of Gd^3+^ ions as G2 resulted in a stable configuration of ions that included two Gd^3+^ ions at the selectivity filter and one Na^+^ ion bound to Site 1b (Fig. S4c). No Gd^3+^ or Na^+^ ion permeation has occurred. G4 simulation contained the highest concentration of Gd^3+^ ions, of which only one initially resided at Site 1. After a short transition period, two Gd^3+^ ions occupied Sites 1a and 1b with similar coordination to G2 (Fig. S4b). Again, no Na^+^ ions or water permeated through the channel. Tight binding of Gd^3+^ ions resulted in nearly complete occlusion of the pore, which is consistent with Gd^3+^ block of TRPV6 channels^36,61^.

## Discussion

In the crystal structure (PDB ID: 5IWP), aspartates D541 coordinate one calcium ion (Site 1), another calcium ion is located in the vicinity of threonines T538 (Site 2), and the third calcium ion is solvated in the central cavity at the level of methionines M569 (Site 3). In our equilibrium MD simulations (e.g. C1 and the second half of the C7.1 trajectory), Ca^2+^ ions shifted from their respective initial crystal structure-like positions, yet remained stably bound within the three binding sites. As illustrated in Fig. 2b, the Site 1 can accommodate two Ca^2+^ ions closely positioned near each other (Sites 1a and 1b). The ion at Site 1a is coordinated by D541 and water from the extracellular vestibule. The ion at Site 1b is coordinated by the D541 oxygen and the backbone oxygen of I539 in the selectivity filter along with water molecules. Position of the third Ca^2+^ ion in the pore is fluctuating between coordinating T538 at Site 2 or water in the central cavity (Site 3). This configuration of ions observed in our MD simulations has significantly lower *ab initio* QM energy, than the configuration found in the crystal structure. This QM result (Fig. S1 and Methods) strongly supports the MD findings. It is possible that due to crystallization conditions and symmetry imposed by the crystals, the upper ion in C1 simulation was stabilized near D541, while the second ion (in the middle of the selectivity filter) occupied a less stable “middle” binding site near T538, which appears to be an intermediate site during ion permeation process. Also, the density around Site 1 seen in crystallographic results may simply be an average of the stable positions at Sites 1a and 1b seen in the simulations.

The Ca^2+^ knock-off permeation mechanism can be described as a two-stage process: i) an incoming Ca^2+^ ion approaches Site 1a from the extracellular vestibule, and ii) the resident Ca^2+^ ion from Site 1b departs to the central cavity. The transition state for this permeation process is characterized by three ions occupying two binding sites (Sites 1a and 1b) formed by D541 residues. The life time of this transition state is determined by the wait time for a consorted fluctuation at two ion positions: the incoming ion motion towards the binding site and the lower resident ion motion towards the central cavity. Simulations C4 and C6 illustrate the second part of the permeation mechanism - transition of the resident ion to the central cavity. In contrast, the C7 trajectory, which starts with a distal Ca^2+^ ion approaching Site 1 from the extracellular vestibule, contains a complete ion permeation event (Supp. Movie 2). Figure 5a–c shows consecutive positions of Ca^2+^ ions during the permeation event and Fig. 5g shows coordinates of all three ions along the channel axis during the trajectory. At the beginning of the trajectory, the upper-incoming ion is fully solvated by water in the ion channel extracellular vestibule (Fig. 5a). The middle and the lower resident ions are bound at Site 1. As soon as the upper ion successfully approaches Site 1a and binds to an oxygen of one of the D541 groups (Fig. 5g), the ion configuration changes dramatically (Fig. 5b). This change in coordination triggers departure of the lower ion, leading to its total solvation in water. In meanwhile, the upper and the middle ions rearrange in such a way that the incoming ion occupies the upper stable position at Site 1a, while the middle ion moves down to the lower stable position at Site 1b (Fig. 5c). Fast water-exchange process, at the rate approaching 10^8^ s^−1^ ^62,63^, results in water molecules absent from Site 1, and no water crosses the channel pore constriction formed by aspartates D541 in presence of ions (see Fig. 6).

It is worth mentioning that the mechanisms of Ca^2+^ and Na^+^ permeation differ drastically. While permeation of Ca^2+^ clearly follows the knock-off mechanism, Na^+^ permeation does not. Many Na^+^ ions occupy the selectivity filter and Site 1 (Fig. 4a), with permeation of the lower ions completely independent of Na^+^ ions approaching from the above, therefore lacking the crucial condition of the Ca^2+^ permeation knock-off mechanism (Supp. Movie 1*)*. The divalent cation permeation sequence for TRPV6 is Ca^2+^ > Ba^2+^ > Sr^2+^ > Mn^2+^^43^. Our simulations demonstrate significant differences in permeation of Ca^2+^ and Ba^2+^. Both ions permeate TRPV6 via the knock-off mechanismbut the observed transition state that includes three ions at Site 1 in both cases has a remarkably different duration. In case of Ca^2+^, it is a short-living state that is often hard to observe. For Ba^2+^, the transition state is long-living and slows down the dynamics of permeation. The main difference between Ca^2+^ and Ba^2+^ in our model comes from their different short-range interactions (Lennard-Jonnes parameters) with the atoms in their first solvations shell, e.g. Ba^2+^ has larger radius but also nearly three times stronger short-range attraction with nearby atoms.

Simulations G2 and G4 suggest that at high Gd^3+^ concentrations, a stable configuration of two Gd^3+^ ions bound at Site 1 blocks the ion channel pore. At lower Gd^3+^ concentrations (G1 simulation), only one Gd^3+^ ion binds at Site 1 but no water or counter ion permeation occurs. Apparently, Gd^3+^ blocks TRPV6 channel at all concentrations. Comparing similar setups for Gd^3+^ (Fig. 7a) and Ca^2+^ ions (Fig. 4b), the major difference is the position of ions at the selectivity filter: Gd^3+^ occupies the region between residues D541 and T538 (Sites 1 and 2, respectively), while Ca^2+^ binds at Site 1 only. Additionally, due to the greater charge of Gd^3+^ ions, Na^+^ ions are effectively repelled from the selectivity filter. As a result, Ca^2+^ allows Na^+^ ions to permeate through the channel, while Gd^3+^ does not.

## Conclusion

We found that at high Ca^2+^ and Ba^2+^ concentrations, three ions resided in the selectivity filter of the TRPV6 channel. The most striking distinction between crystal structure and simulations is that two instead of one ion prefer to interact with the ring of aspartates D541 at the selectivity filter. The equilibrium positions of these ions differed slightly from the positions observed in TRPV6 crystal structures. Specifically, Site 1 accommodated two ions in the simulations (sites 1a and 1b), suggesting that the single ion positioning in the crystal structures may be a result of averaging over the two closely spaced and thus indistinguishable positions in crystallographic densities. The differences in the crystallographic and simulation conditions, such as temperature, salt concentrations and the presence or absence of the lipid bilayer, may as well play a role in the observed different binding of ions in the selectivity filter. Consistent with physiological experiments, Na^+^ permeates the selectivity filter in the absence of divalent cations. At low Ca^2+^ concentrations, Ca^2+^ ion bound to Site 1 does not preclude Na^+^ from permeating the channel. At high Ca^2+^ concentrations, Ca^2+^ permeates according to the knock-off mechanism. No water permeates the channel constriction together with the ions, while ions are partially dehydrated when bound to Site 1. Ba^2+^ also permeates via the knock-off mechanism but does it slower. Gd^3+^ binds tightly at the selectivity filter, blocks the channel and prevents Na^+^ from permeating the pore. Further studies are required to compute relative energetics of various ions coordination at the binding site, and also, an open channel model is needed to directly simulate the process of ion permeation through the entire TRPV6 channel in open conformation.

It is important to note in conclusion that modeling of protein interactions with the divalent and, especially tri-valent cations remains an area of active research in the field of computational chemistry. While Ca^2+^ models are fairly well developed and limitations of various approaches are well understood, modeling of the trivalent Gd^3+^ ion is a relatively novel attempt. Therefore, the model of Gd^3+^ behavior has to be taken cautiously. Further work is needed to develop Gd^3+^ ion models that account for its complicated quantum mechanical nature and high polarizability and polarizing potential. Despite this potential limitation of the method we believe that the leading force of this blocker ion interaction with the protein is due to its relatively small size and high charge. This resulted in an occupancy of the entire binding site by a single ion or by two ions.

## Methods

### Protein system model

The initial atomic coordinates for simulated proteins were taken from the corresponding X-ray structures (5IWK, 5IWP, 5IWR, and 5IWT for Apo, Ca^2+^, Ba^2+^, and Gd^3+^ bound states of TRPV6, respectively(23)) and contained a tetramer of the pore helices (S5, P, and S6) and a fragment of the TRP helix, from K483 to Q595. AmberTools Leap^64^ was used to construct simulation boxes, which consisted of a bilayer of 567 lipids for Apo system, 566 lipids for Ba^2+^ system, and 562 lipids for Ca^2+^ and Gd^3+^ systems. The box also contained 32181, 33166, and 33162 TIP3P water molecules for Apo, Ba^2+^, and Ca^2+^/Gd^3+^ systems, respectively. The titratable residues were set to the most likely protonation state at neutral pH, and histidine residues protonated in ε position. The charge of all simulated systems was set to neutral by adding Na^+^ ions. A representative equilibrated system with Na^+^ and Ca^2+^ ions in the simulation box is shown in Fig. 1.

### Molecular Dynamics simulations

#### Trajectory generation

Crystal structure-based models of TRPV6 were first equilibrated according to the procedure described in the next section. The Apo and Ca^2+^-, Ba^2+^-, or Gd^3+^-containing models were used to perform relatively short equilibrium simulations CA, C1, B1 and G1, respectively (Supplementary Table ST1). The number and placement of the ions were maintained as in the corresponding crystal structures. The trajectories performed and described in this work are shown in Supplementary Scheme Sc1. From each of these trajectories we have extracted the most stable structure and the structure with the lowest root mean square deviation (RMSD) from the corresponding crystal structure. The lowest RMSD structures also had the lowest fluctuations of the selectivity filter, including D541. The average RMSD for the channel are shown in the Supplementary Table S2. The channel remained closed and stable during all simulations (see Fig. S6 for RMS Fluctuations). Throughout simulations, the loop between T538 and D541 remained relatively stable with the relevant side chains bound to respective ion. The loop above D541 was more mobile than the rest of the protein. Due to its mobility, the volume of the channel vestibule also varied. The channel vestibule is defined as the space above D541 (binding Site 1) and up to the recruitment sites on the top of the helix connecting loops, which host the ion recruitment sites (Fig. 2a).

#### Protocols for all MD simulations

AMBER14^64^ software package was used for all Molecular Dynamics (MD) simulations. AMBER99SB-ILDN^65^ force field was used for the protein and ions, TIP3P model for water^66^, and Amber Lipid14^67^ model for lipids. For constant temperature simulations, temperature was controlled using Langevin thermostat and for constant pressure simulations, the pressure was controlled using Berendsen barostat with anisotropic pressure scaling. The electrostatic interactions were approximated using Particle Mesh Ewald (PMEMD)^68^ and its implementation in CUDA. The non-bonded interactions cutoff radius was 8.0 Å. Covalent bonds involving hydrogen atoms were constrained using SHAKE^69^, which allowed to use the time step of 2 fs. The trajectories were post-processed with VMD^70^, CPPTRAJ^71^, and Pymol^72^ software packages.

#### Protein MD Equilibration protocol

The systems of TRPV6 in apo, Ca^2+^-, Ba^2+^-, and Gd^3+^-bound states were created using Leap and equilibrated as follows. The first short minimization was performed focusing on water and lipid molecules. The second stage consisted of NVT ensemble simulations, with the temperature of the system increasing, was performed in 5 steps with following parameters: protein and water molecules constrained with harmonic force constant k = 10 kcal/mol/Å^2^ for 100 ps and temperature (T) increasing 0.1–100K, protein and water constrained with k = 5 kcal/mol/Å^2^ for 100ps and T increasing 100K–150K, protein and water constrained with k = 2 kcal/mol/Å^2^ for 100ps and T increasing 150K–200K, only protein constrained with k = 2 kcal/mol/Å^2^ for 100ps and T increasing 200K–300K. The final stage of equilibration consisted of NPT simulations at T=300K while gradually releasing protein constrains over 25 ns.

### *Ab Initio* Quantum Mechanics Calculations

*Ab Initio* quantum mechanics (QM) calculations of energy of clusters of Ca^2+^ with water and Asp residues were performed using Hartree-Fock method (HF) with 6–311+(d,p) basis set. Calculations with the higher level MP2 method and a basis set with larger number of diffuse functions resulted in similar computed relative energies of the ion-ligand clusters. Single point energy calculations were performed on *ca*. 310 clusters of 2 Ca^2+^ ions surrounded by 4 D541 residues and 8 water molecules for each of the two stable configurations extracted from MD simulations (Fig. S1). Additional background point charge distribution was included. The point charges were imported from the charges of atoms of molecules in MD simulations surrounding the Ca^2+^ ions in the selectivity filter within a sphere of 12 Å radius^73^.

### Data Availability

All data generated or analyzed during this study are available from the corresponding author on reasonable request.

## Electronic supplementary material

Supplementary information

Movie 1. Na+ permeation through TRPV6 channel

Movie 2. Knock-off mechanism of Ca2+ permeation
