# Supplementary material for: Ion Permeation Mechanism in Epithelial Calcium Channel TRVP6
Source: Sci Rep. 2018 Apr 9;8:5715. doi: 10.1038/s41598-018-23972-5 (PMC5890290; doi:10.1038/s41598-018-23972-5)
Supplement: Supplementary file 1 — Supplementary information [file 41598_2018_23972_MOESM1_ESM.docx]

Supplementary Information

(Supplementary Figures 1-6, Supplementary Tables 1-3, Supplementary Scheme 1, and Supplementary Movies 1, 2)

Ion Permeation Mechanism in Epithelial Calcium Channel TRVP6

# Serzhan Sakipov, Alexander I. Sobolevsky, and Maria G. Kurnikova

# Figures


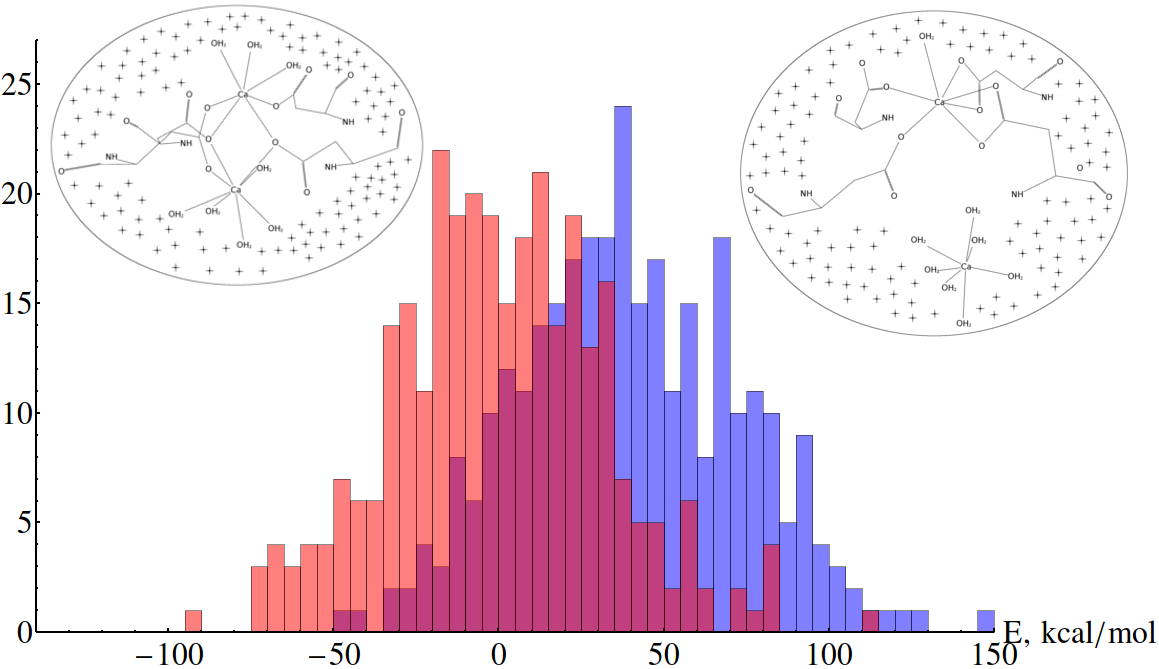


**Figure S1. Ab initio quantum mechanical energy calculated for two Ca^2+^ ions bound at Site 1.** All structures for QM calculations were extracted from equilibrium MD simulations. Two Ca^2+^ ions and their coordinating chemical groups included in QM calculations are shown as inserts. All other atoms within 12Å distance were included as partial charges, shown in the inserts as crosses (see Methods for details). Blue bars correspond to the crystal structure-like ion configurations (shown schematically in the right insert). Red bars correspond to structures obtained from equilibrium MD simulations (Fig. 1B; shown schematically in the left insert). Zero energy is set to the average energy of the clusters from the equilibrium MD simulation. The average value of the energy for the crystal structure-like clusters equals to 39.5 kcal/mol.

**
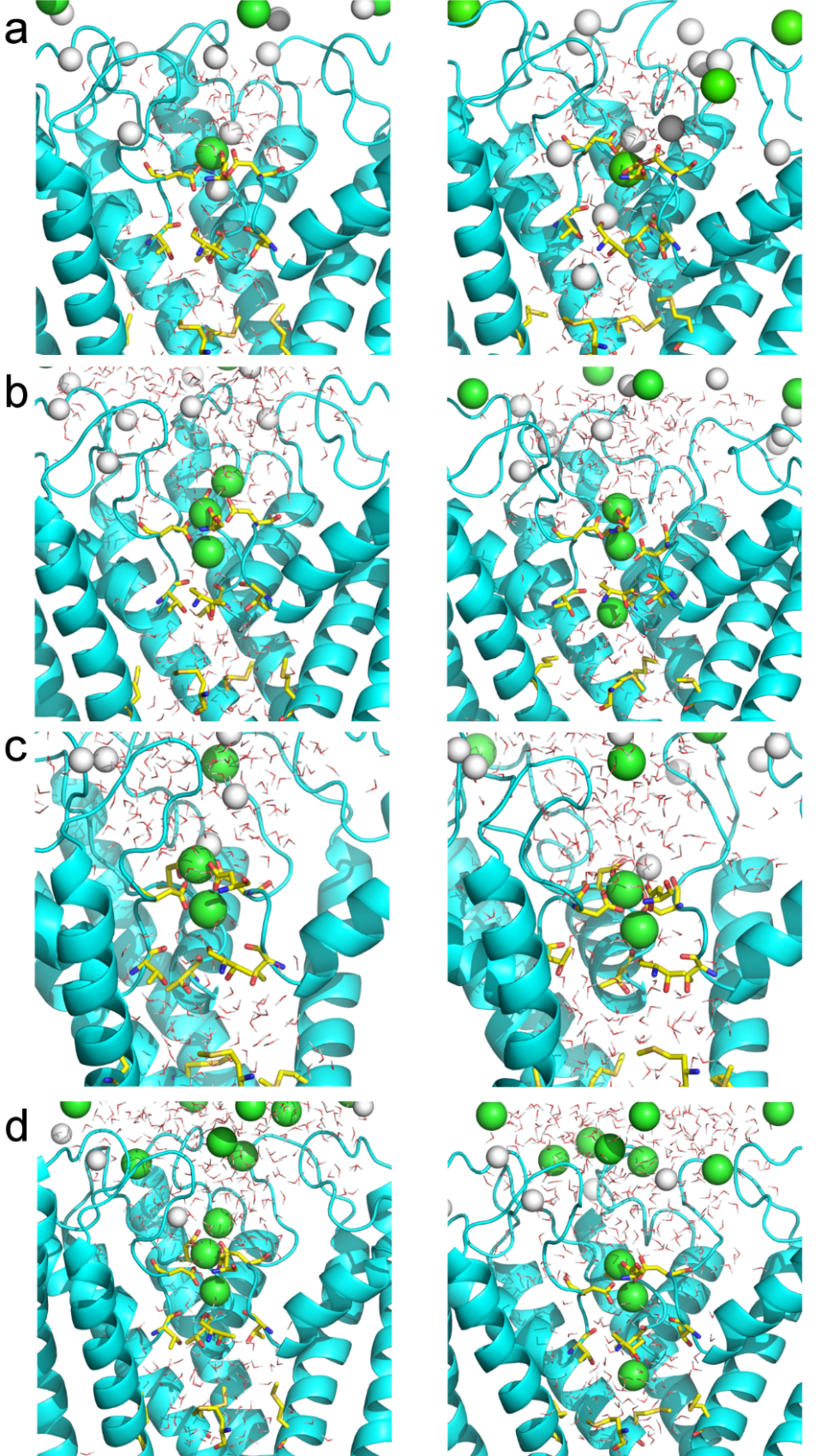
**

**Figure S2. Initial and final configurations of Ca^2+^ ions in the non-equilibrium simulations**. Left and right columns correspond to the initial and final configurations of ions for each of the simulation: C3(a), C4(b), C5(c), and C6(d). Protein is shown in cyan. Water is in wireframe representation. Ca^2+^ and Na^+^ ions are shown as green and white spheres. The residues surrounding binding sites are shown in yellow.

| a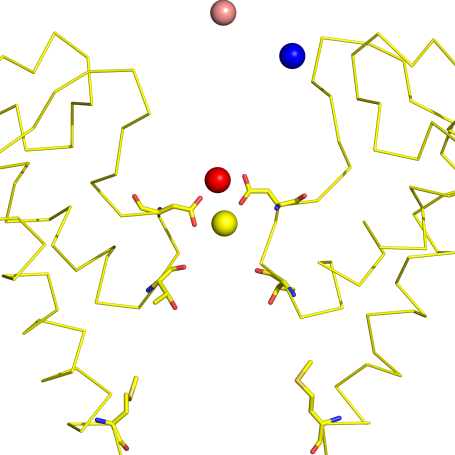 | b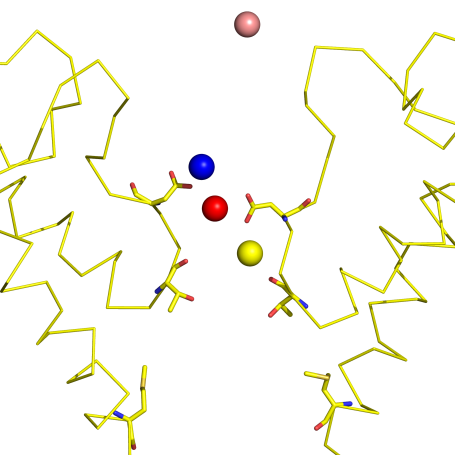 | c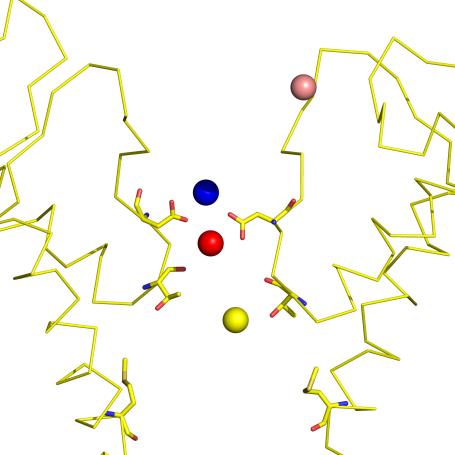 |
| --- | --- | --- |
| d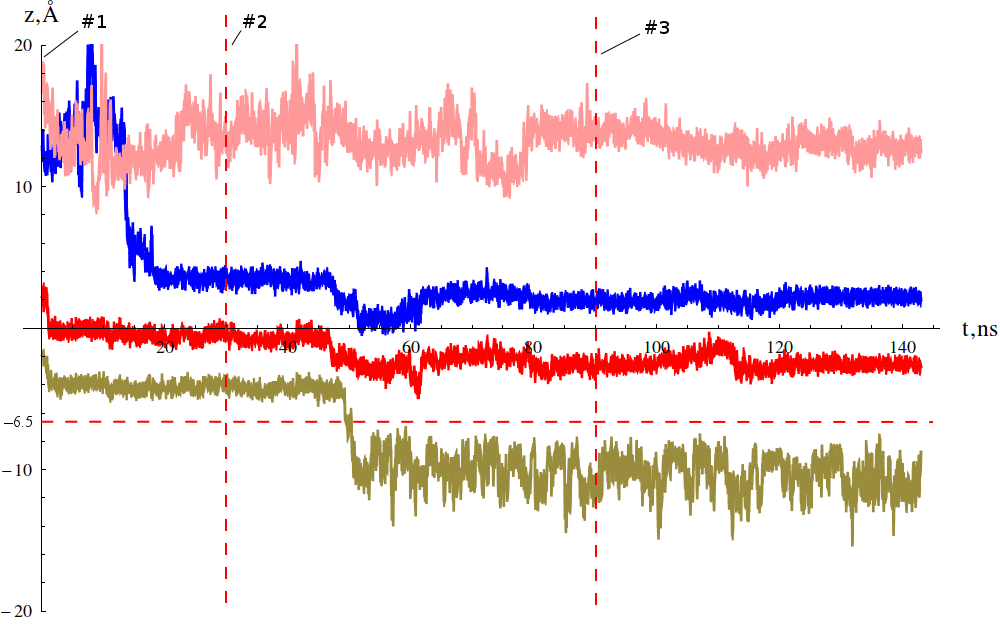 | | |

**Figure S3. Mechanism of Ba^2+^ permeation through the TRPV6 channel**. (a)-(c), Sequential configurations of Ba^2+^ ions during B2 simulation: (a) an initial configuration, (b) a transition state, and (c) a final configuration. The blue, red and yellow spheres represent incoming, intermediate and leaving Ca^2+^ ions, respectively. The pink sphere shows the fourth Ba^2+^ ion closest to D541. Residues D541, T538, and M569 are shown in stick representation. (d), Positions of ions along the z-axis during the B2 simulation. The three states #1, #2, and #3 occurring at the times indicated by vertical lines correspond to the ions configurations shown in (a), (b), and (c), respectively. The horizontal red dashed line indicates the level of ion crossing into the channel.

| a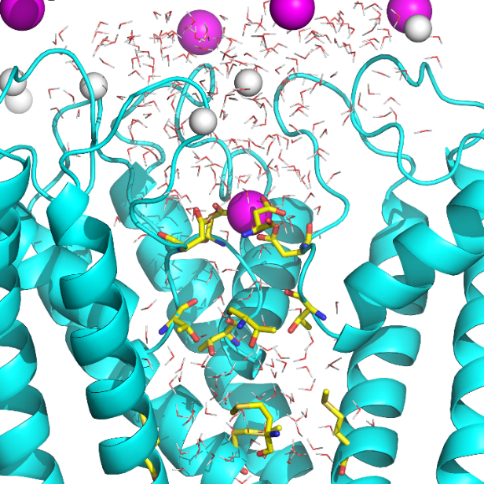 | d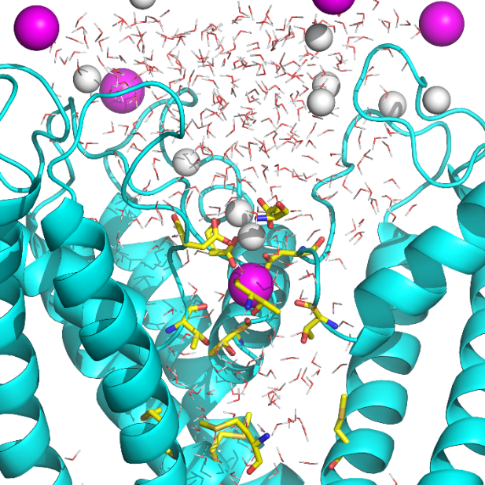 |
| --- | --- |
| b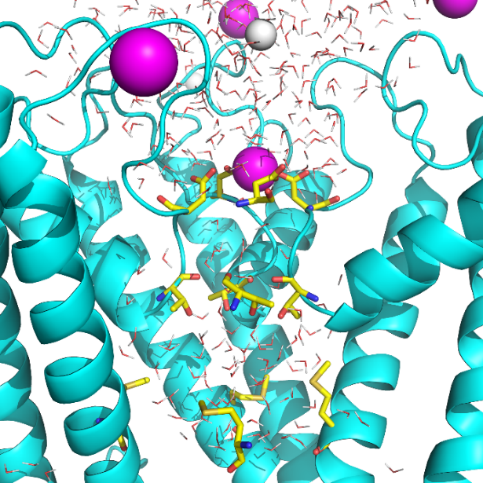 | e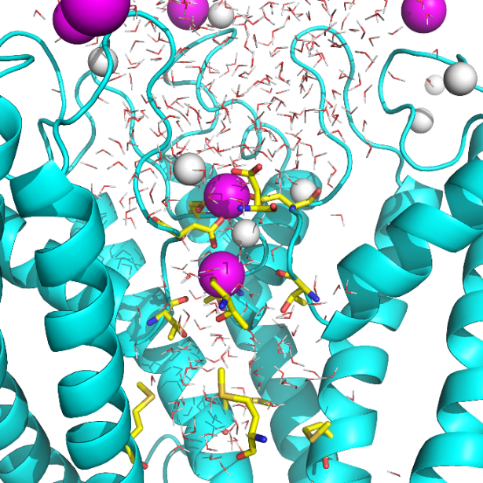 |
| c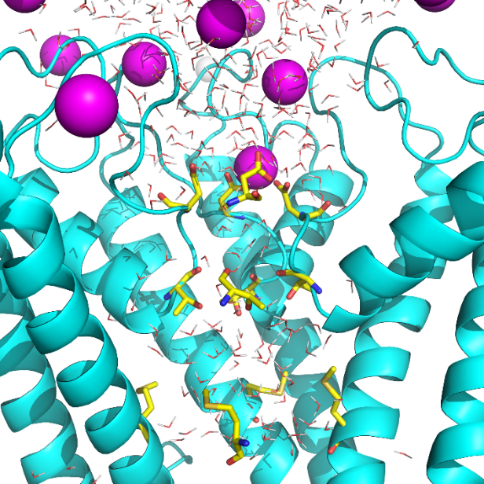 | f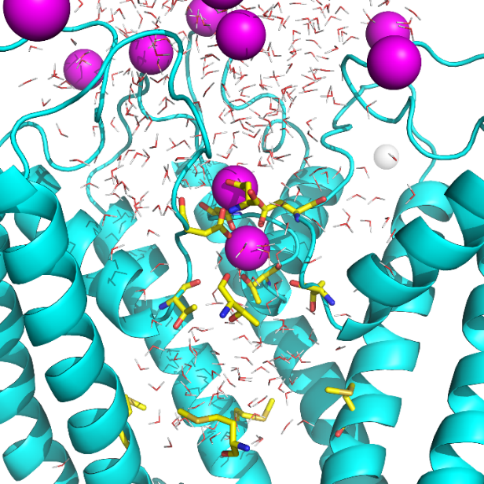 |

**Figure S4. Initial and stable configurations of Gd^3+^ ions.** Left and right panels represent the initial and stable configurations of Gd^3+^ ions in (a) G1, (b) G3, and (c) G4 simulations. The stable configurations persisted for the last 25 ns of each simulation. Gd^3+^ and Na^+^ ions are shown as pink and white spheres. Protein is shown in cyan. Water is in wireframe representation.

| a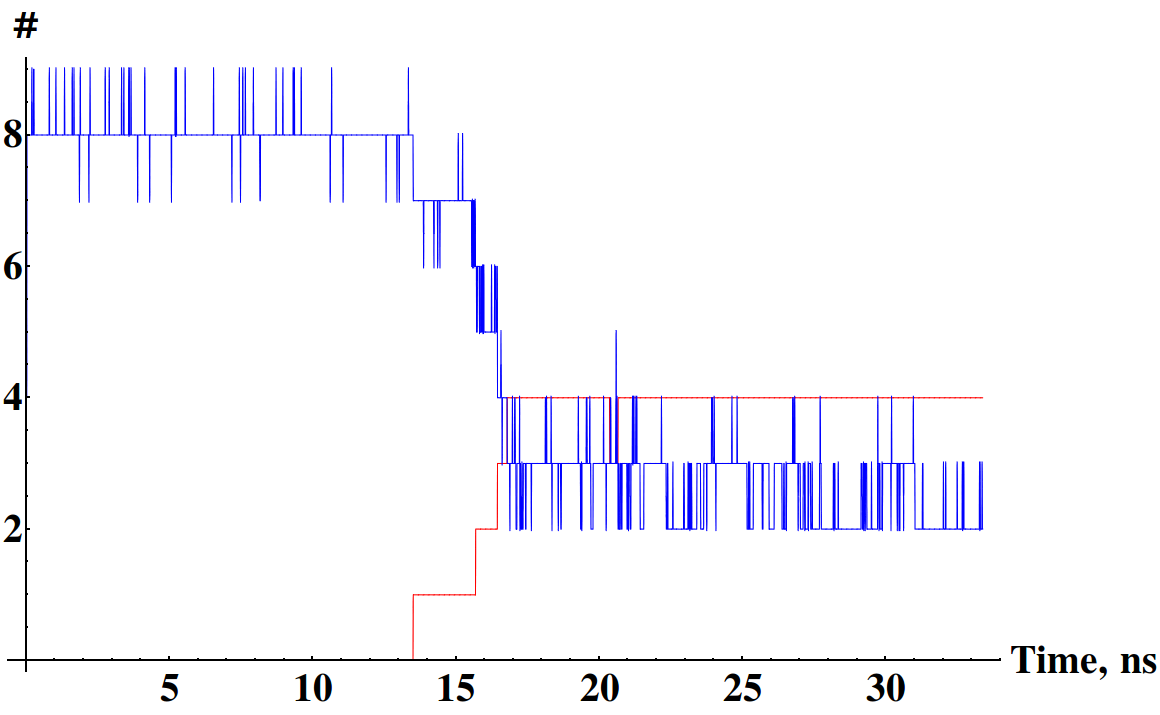 |
| --- |
| b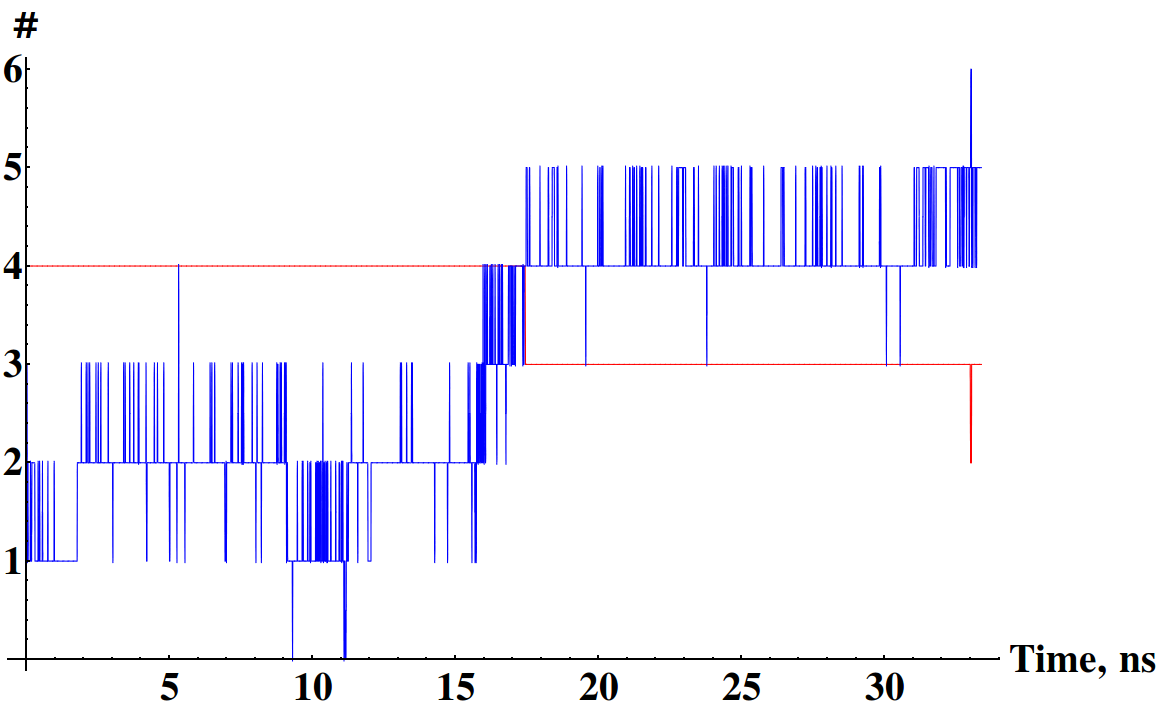 |
| c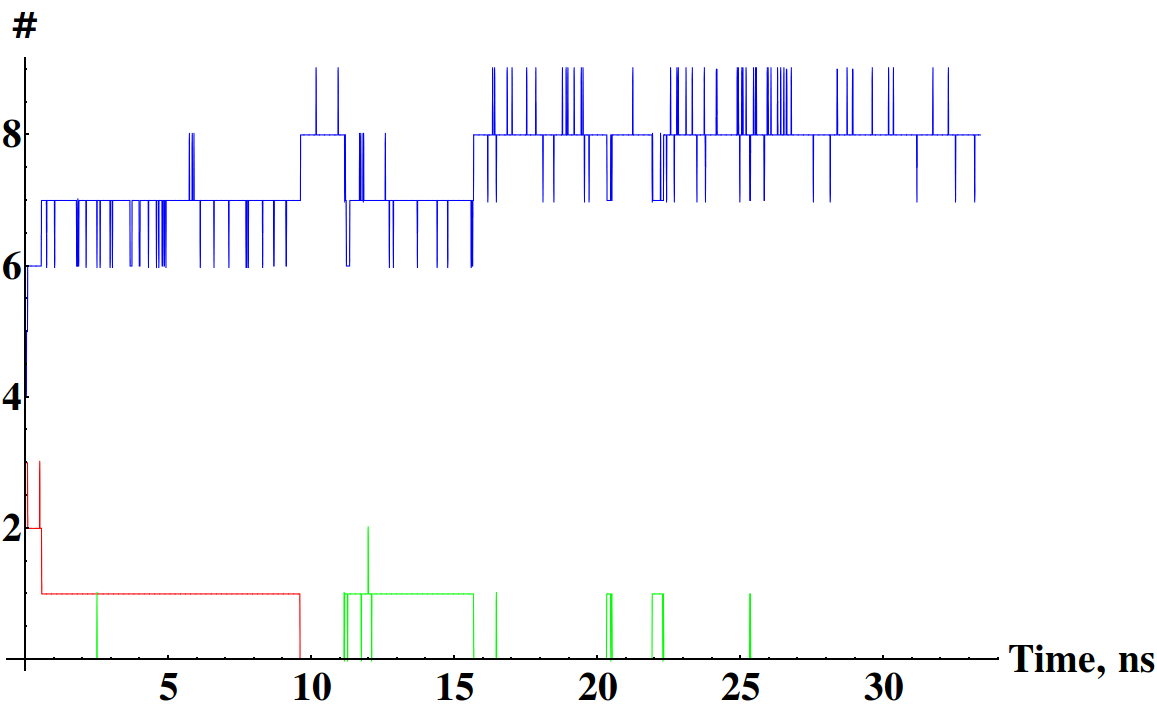 |

**Figure S5.** **Plots of the number of D541, T538, and water molecules coordinating Ca^2+^ ions during permeation**. Red, green, and blue lines represent coordination with D541, T538, and water molecules for (a) incoming, (b) middle, and (c) leaving Ca^2+^ ions in C7.1 trajectory.

**
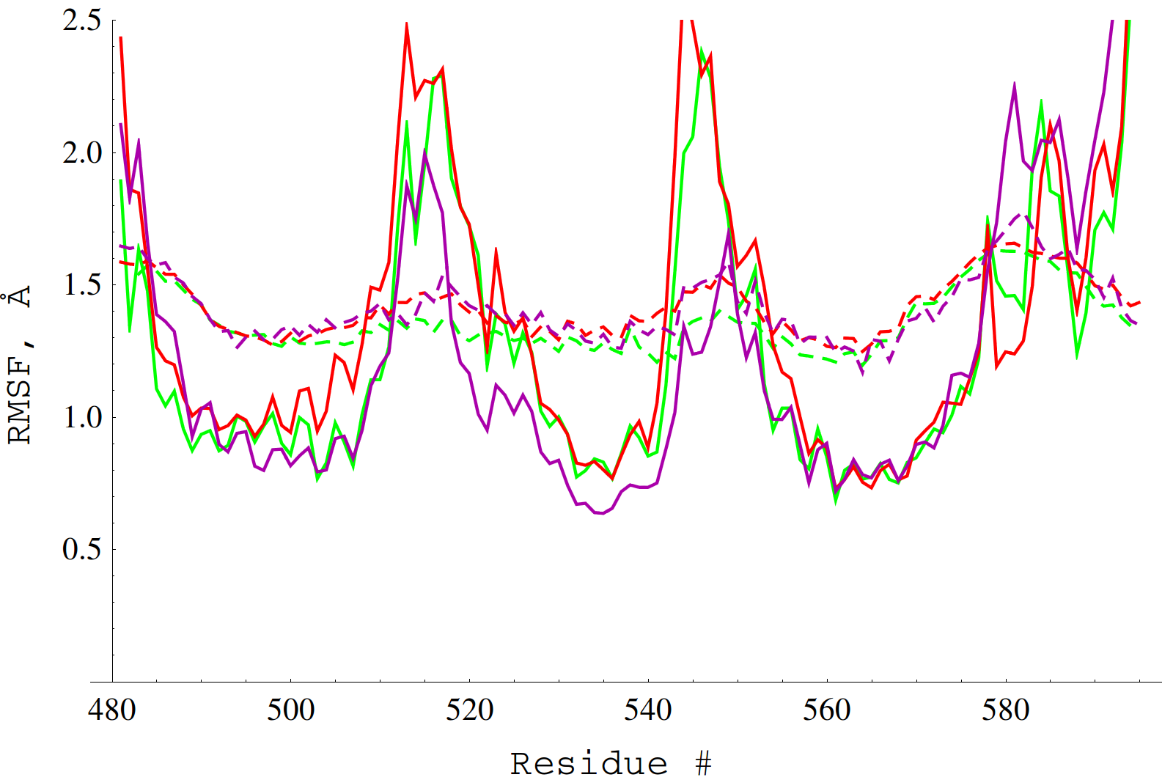
**

**Figure S6. RMS Fluctuations for Cα atoms.** RMSF values were calculated for the trajectories C5, B2, and G2 (solid green, red, and magenta lines, respectively), and for the experimental B-factors in structures with Ca^2+^, Ba^2+^, and Gd^3+^ (dashed green, red, and magenta lines, respectively). For the pore domain, the residue boundaries are I481 to Q512 for S5, Y525 to F536 for the P-loop, and Y554 to M577 for S6. Ca^2+^ binds at D541. RMSF values for residues in helices are below 1.0 Å, while for the connecting loops above 1.5 Å. High RMSF values for residues after M577 reflect possible TRP helix unfolding, which was truncated in order to stabilize the S6 helix. Similarly, high fluctuations at the other end of the pore, before I480 in helix S5, indicates possible unfolding of the N-terminal portion of S5. Note, fluctuations of residues in the P-loop helix and the selectivity filter loops are the lowest, indicating their stability. Fluctuations for the C5 and B2 trajectories are overall higher than for G2.

# Tables

**Table ST1.** List of MD trajectories. Presented are the numbers and locations of Na^+^ and multivalent ions for the apo trajectory (CA) and trajectories with multivalent metal ions, including Ca^2+^ (C1 through C8), Ba^2+^ (B1 and B2), and Gd^3+^ (G1 through G4).

| Trajectory  Name | # of Na^+^ | | # of Metal ions | | | Time, ns | Position of Ca^2+^/Ba^2+^/Gd^3+^ |
| --- | --- | --- | --- | --- | --- | --- | --- |
| CA | | 34 | | Ca^2+^ | 1 | 10* | 1 at D541 site |
| C1 | | 29 | |  | 3 | 24 | 1 at D541 site, 1 at T538 site, 1 in cavity |
| C2 | | 36 | |  | 0 | 31 |  |
| C3 | | 26 | |  | 5 | 120 | 4 at recruitment sites, 1 at D541 site |
| C4 | | 22 | |  | 7 | 36 | 4 at recruitment sites, 3 at D541 site |
| C5 | | 20 | |  | 8 | 160 | 4 at recruitment sites, 2 in vestibule, 2 at D541 site |
| C6 | | 14 | |  | 11 | 30 | 4 at recruitment sites, 4 in vestibule, 3 at D541 site |
| C7.1 | | 14 | |  | 11 | 21 | 4 at recruitment sites, 5 in vestibule, 2 at D541 site |
| C7.2 | | 14 | |  | 11 | 35 | 4 at recruitment sites, 5 in vestibule, 2 at D541 site |
| B1 | | 22 | | Ba^2+^ | 7 | 17* | 4 at recruitment sites, 1 at D541 site, 1 at T538 site, 1 in cavity |
| B2 | | 20 | |  | 8 | 143 | 4 at recruitment sites, 2 in vestibule, 2 at D541 site |
| G1 | | 21 | | Gd^3+^ | 5 | 76* | 4 at recruitment sites, 1 at D541 site |
| G2 | | 16 | |  | 8 | 160 | 4 at recruitment sites, 2 in vestibule, 2 at D541 site |
| G3 | | 16 | |  | 8 | 27 | 4 at recruitment sites, 1 in vestibule, 3 at extracellular region |
| G4 | | 3 | |  | 11 | 36 | 4 at recruitment sites, 4 in vestibule, 3 in extracellular region |

*only production run included

**Table ST2.** Root mean square deviations (RMSDs) for Cα atoms in S5, P-loop and S6 and for the entire protein (Tot). CA, C1, B1, and G1 are short trajectories starting with crystal structures in apo, Ca^2+^-, Ba^2+^-, and Gd^3+^-bound states. C5, B2, and G2 are long trajectories starting from the corresponding stable structures (see text). RMSD values are below 1.9 Å for S6 and 1.6 Å for the P-loop, while ~3 Å for S5. In crystal structures, S5 is enveloped with helices S1, S3, and S4. In contrast, S5 in simulations is exposed to membrane and its interaction with lipids might cause destabilization of this helix. Nevertheless, during simulations, the entire S5 maintained its helical structure and its relative positioning remained stable (see *Table S3* for deviations in tilt angles of the helices).

| Helix | CA | C1 | B1 | G1 | C2 | B2 | G2 |
| --- | --- | --- | --- | --- | --- | --- | --- |
| S5 | 1.2 | 1.8 | 1.6 | 2.5 | 3.4 | 2.9 | 2.7 |
| P | 0.9 | 1.2 | 0.9 | 1.1 | 1.6 | 1.4 | 1.5 |
| S6 | 1.3 | 1.5 | 1.4 | 1.5 | 1.9 | 1.8 | 1.7 |
| Tot | 2.0 | 2.6 | 2.1 | 3.0 | 3.3 | 3.1 | 3.3 |

**Table ST3.** Angles of helices relative to Z axis. The angles in degrees are listed for the C2, B2 and G2 trajectories. Deviations for all angles are below 2.7 degree, and the angles for the same helices are the same across the three trajectories.

| Helix | C2 | B2 | G2 |
| --- | --- | --- | --- |
| S5 | 66.6 ± 2.1 | 68.0 ±1.1 | 65.0 ±2.7 |
| P | 54.6 ±1.2 | 56.6 ±0.9 | 54.5 ±0.9 |
| S6 | 40.0 ±1.9 | 41.8 ±1.2 | 39.4 ±1.1 |

Apo

Ca^2+^

Ba^2+^

Gd^3+^

CA

C1

B1

G1

Stable

Low RMSD

B2

C2

C3,4,5,6,7

G2

G3,4

PDB crystal structure

Stable structure with the lowest RMSD to PDB

Ba^2+^

Ca^2+^

Gd^3+^

Equil

Equil

Equil

Equil

**Scheme Sc1.** Scheme of trajectory generation. Left four blocks represent the crystal structures obtained from PDB (5IWK, 5IWP, 5IWR, and 5IWT for apo, Ca^2+^-, Ba^2+^-, and Gd^3+^-bound structures). The CA, C1, B1, and G1 blocks represent trajectories run from crystal structures. The block in the middle represents stable conformations of TRPV6 with the lowest RMSD compared to the corresponding crystal structures. The blocks on the right represent the trajectories run for each of the corresponding ions.

# Movies

**Movie 1. Na^+^ permeation through TRPV6 channel.** Protein is shown in cyan cartoon representation, Na^+^ ions are shown as white spheres, and D541, T538, M569 are shown as yellow sticks with oxygen and nitrogen in red and blue respectively. Multiple Na^+^ ions occupied the selectivity filter. The total length of the simulation shown is ca. 30 ns.

**Movie 2. Knock-off mechanism of Ca^2+^ permeation.** Protein is shown in cyan cartoon representation. The incoming, intermediate, and leaving Ca^2+^ ions are shown as blue, magenta, and brown spheres respectively, other Ca^2+^ ions are show as green spheres. D541 and T538 are shown as yellow sticks with oxygen and nitrogen in red and blue respectively. Ca^2+^ permeates via knock-off mechanism. The total length of the simulation shown is ca. 30 ns.
